# Stance4Health Nutritional APP: A Path to Personalized Smart Nutrition

**DOI:** 10.3390/nu15020276

**Published:** 2023-01-05

**Authors:** Daniel Hinojosa-Nogueira, Bartolomé Ortiz-Viso, Beatriz Navajas-Porras, Sergio Pérez-Burillo, Verónica González-Vigil, Silvia Pastoriza de la Cueva, José Ángel Rufián-Henares

**Affiliations:** 1 Centro de Investigación Biomédica, Departamento de Nutrición y Bromatología, Instituto de Nutrición y Tecnología de los Alimentos, Universidad de Granada, 18071 Granada, Spain; 2 Instituto de Investigación Biosanitaria ibs.GRANADA, Universidad de Granada, 18071 Granada, Spain; 3 Departamento de Ciencias de la Computación e Inteligencia Artificial, Universidad de Granada, 18071 Granada, Spain; 4 Gestión de Salud y Nutrición S.L., 33003 Oviedo, Spain

**Keywords:** computational nutrition, meal plan generator, nutritional app, nutritional intervention, smartphone application, diet app, diet record

## Abstract

Access to good nutritional health is one of the principal objectives of current society. Several e-services offer dietary advice. However, multifactorial and more individualized nutritional recommendations should be developed to recommend healthy menus according to the specific user’s needs. In this article, we present and validate a personalized nutrition system based on an application (APP) for smart devices with the capacity to offer an adaptable menu to the user. The APP was developed following a structured recommendation generation scheme, where the characteristics of the menus of 20 users were evaluated. Specific menus were generated for each user based on their preferences and nutritional requirements. These menus were evaluated by comparing their nutritional content versus the nutrient composition retrieved from dietary records. The generated menus showed great similarity to those obtained from the user dietary records. Furthermore, the generated menus showed less variability in micronutrient amounts and higher concentrations than the menus from the user records. The macronutrient deviations were also corrected in the generated menus, offering a better adaptation to the users. The presented system is a good tool for the generation of menus that are adapted to the user characteristics and a starting point to nutritional interventions.

## 1. Introduction

Healthy eating is one of the most important health challenges in the current global context due to its role in disease prevention [1,2,3]. Unhealthy diet is one of the main risk factors for several diseases, being responsible for some 14 million deaths each year [4]. Adherence to a high quality diet or a prudent dietary pattern has been reported in several studies to be inversely associated with a reduced risk of mortality [1,5]. Therefore, countries are trying to promote healthy lifestyles by developing dietary and health guidelines and recommendations [5]. One of the main difficulties found is that individual responses to dietary advice and intervention are heterogeneous, which shows the need to develop precision or personalized nutrition (PN) for each individual [6]. There are different “levels” of customization, from simple questionnaires to personalized supplements [2]. PN involves many factors, such as nutritional intake, physical activity, individual characteristics specific to each person, dietary advice, dietary products and supplements, health biomarkers, gut microbiota composition and even genetic load [2,6]. All this helps to understand and create a personalized nutritional guide for each individual. Although PN is still a relatively young phenomenon, such is the interest that the European Union (EU) has generated a regulatory framework around PN [2]. PN has great potential for disease prevention, especially when combined with the power and accessibility to mobile technology [7].

Advances in technology have led to an ever-increasing use of electronic devices such as tablets and mobile phones. More than five thousand million people around the world are estimated to own mobile devices, and more than half of them are smartphones [8]. Not only are adults using these devices, but children and teenagers are becoming the main users [9]. For example, digital technologies play an important role in young people’s daily lives and are used to search for relevant information such as health, medicines or drugs [10]. One of the main advantages of these devices are the mobile applications (APPs, which are used for a multitude of things (games, shopping, public services and other functions) [4]. There is currently a growing interest in mobile health applications (mHealth) for health promotion and prevention of chronic diseases. mHealth has great potential, especially in terms of cost-effectiveness and innovation [4,11,12]. Currently, there are an estimated 300,000 mobile health applications available, of which around 10,000 are about nutrition and diet, and some of them are very popular in terms of user downloads [13,14,15]. Diet-related APPs ranked second in the ‘health and fitness’ category of the Google Play Store in 2020 in countries such as Italy [14]. However, it is also worth noting that, due to the overload of nutritional APPs and its possible economic revenue, it is key to differentiate between those that have a scientific basis and those that do not. Not all APPs available to consumers are always reliable, useful and of high quality, nor are health professionals or researchers involved in their creation. Most of the time, a bad approach can have counterproductive effects on health or lead to poor eating habits.

In terms of how diet and nutrition-focused APPs work, their approaches are very varied: some are able to record dietary intake and physical activity, others try to score and compare foods; few APPs are able to generate a meal plan or a healthy shopping list; some are even able to set goals or receive continuous feedback on dietary behavior [1,15,16,17]. These APPs can not only improve diet and make it healthier, but they can also help people with nutrition-related diseases, such as obesity, food allergies or intolerances [14,18]. APPs can offer more possibilities due to artificial intelligence (AI), which is essential for understanding complex biological phenomena [15,19], and combined with the use of big data and machine learning can benefit PN [6]. APPs-based interventions have been shown to be effective in improving diet and the results are comparable to those of traditional non-digital interventions [12]. They also increase follow-up rates and user motivation [5,18].

These APPs have several advantages over conventional methods: they are not dependent on respondents’ memory since they register food in real time [20], and they are based on portable devices and have a better social acceptance [19,21]. In research, they also help to decrease workload, and reduce time and the risk of transcription errors [17,19]. However, there are some limitations, mostly concerning data accuracy, limited nutrient or food data, subjectivity in the scales provided or in estimating portion sizes and digital illiteracy [21,22]. Therefore, APPs are often supported by different chemical, electrical or physical sensors [6,15,19]. Mobile and portable sensors are non-invasive and are able to monitor variations and provide real-time guidance [6,19]. Examples include sensors that measure glucose levels or sweat compounds [6], using the camera as a barcode reader to recognize food labels or algorithms for automatic recognition of food portions from pictures [19]. Wearables such as wristbands or smartwatches are the most popular health monitoring devices, whose technology has advanced quickly in recent years [15]. Due to their feasibility, cost-effectiveness and immediacy of data collection, personalized nutrition APPs combined with emerging technologies or wearables can be an effective method in nutritional interventions [8,13,19,23].

Therefore, one of the goals of the Stance4Health (Smart Technologies for personAlised Nutrition and Consumer Engagement, S4H) project [24], financed by the European Union’s Horizon 2020 program, is to create and offer, with the help of new technologies and rigorous scientific support, a PN service through the use of an APP. This study shows the APP created in the framework of the project after a close collaboration between APP developers, nutritionists and scientists from different areas, all in order to create a rigorous and quality APP. This APP is under use in a multi-country nutrition intervention for adults and children (Trial ID: ISRCTN63745549) [25]. The APP aims to promote balanced nutrition and healthy habits by creating personalized recommendations for each individual. The APP not only takes into account personal characteristics, activity factors and dietary preferences, but it also takes into account gut microbiota nutritional requirements.

## 2. Materials and Methods

### 2.1. Development and Characteristics of the S4H APP

**Figure 1:**
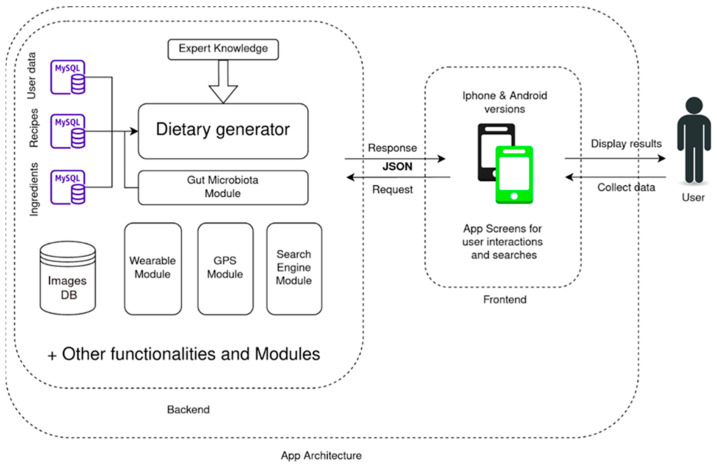
Overview of the S4H APP design and architecture.

The S4H APP was developed under the framework of the Stance4Health European project and aims to support human nutrition trials in several countries [25]. Personalized nutritional systems usually manage different sources of information that could serve for different purposes. In addition, all that information has to be stored in a proper design, as we should be able to upload, retreat or update it at any point. Those reasons led us to build the S4H APP following a modular design, where modules can interact with different sources of information and between them. However, at the same time, these modules can be changed or updated without disrupting the APP functioning. In Figure 1, we represent a global schema of the APP, which we will use as a summary of it. The diagram represents both the server-side of the application (backend) and the user-side (frontend). The backend is where all modules are stored and where all the calculations are made. The dietary generator is the main module, which receives information from the experts, what the users need, different recipes and ingredients (stored in independent databases). Moreover, if there is information about microbiomes, it can be used to modify the generation, too. Another 4 modules are represented, as they constitute other functionalities that can be used within the APP; however, for clarity, not all the links between modules and databases are drawn. All these modules are running in the servers, with the information stored in the databases and the data collected with the APP. The frontend is the visual side of the APP, that which is seen by users; it is used to show the user (in a comprehensible way) the data produced by the different modules. In addition, it also collects the changes from the users and sends it to the backend (to be stored in the database and considered when specific modules are activated).

### 2.2. APP Architecture

We divided the APP framework between the frontend and the backend. An overall view of the APP architecture and main modules are depicted in Figure 1.

**Figure 2:**
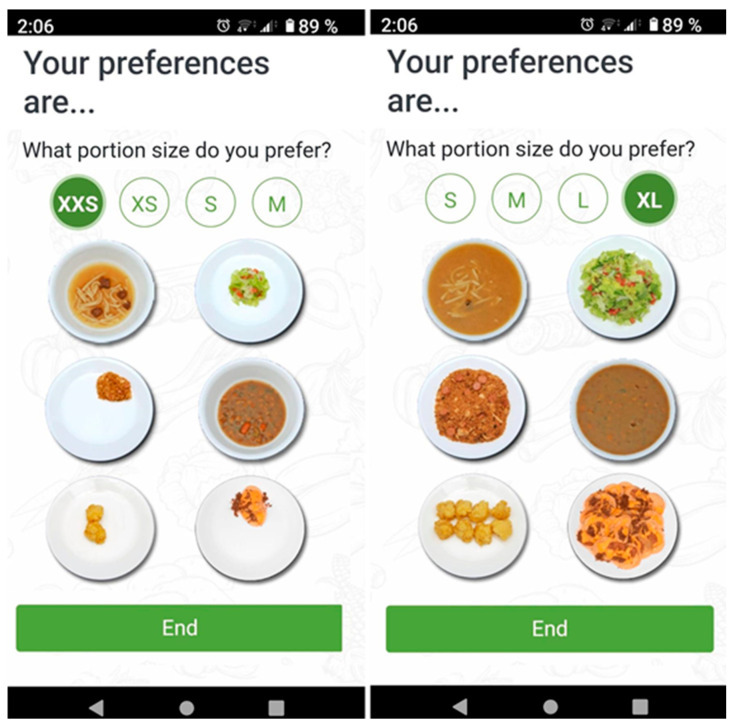
XXS and XL portion sizes of the APP.

The frontend consists of all the services the user either sees or interacts with when they open the APP. It is responsible for the global look and feel of the APP experience and our main source of input data. The main parts of the frontend are the screens. The term “screen” refers to the different graphical displays associated with every module that the users see navigating through the APP (for example, screens depicted in Figure 2). Screens collect the changes and choices of the user and sent them to the backend. They do it in a machine-readable format that it is easily stored in the databases. Later, this data will be used when any of the backend modules need it. In our case, this whole section is built by Angular 8 and Bootstrap 4 which were used to design and create this side of the APP.

The backend or server-side section was built by all the technology that the user does not directly see or interact with. In our case, it was built with a modular design, which lets us connect or disconnect different additional functionalities to the APP. In Figure 1, a diagram of this part is also presented. The backend is first constituted by the databases. Those are represented in blue in the diagram, and they are the main source of information of the system. There, we store all the user’s data and changes as well as all the nutritional information of the recipes. All these data are submitted to the dietary generator module to produce a dietary recommendation. This led us to the second type of element that appears in the backend: the modules. Modules are a collection of instructions or functionalities that can be activated while the user uses the app; for example, the search module is activated when the user accesses the “recipe search’s screen”; it gathers the text the user is looking for, get the data from the databases that matches it, and send the results back to the screen, so the user can see them in a graphical way. It is worth noting that some of the modules represent independent functionalities, so they can receive and produce information without affecting the others (i.e., a recipe search is independent of the data received by the GPS module). A further description on the interaction between modules can be read in Section 3.1. 

On the technical side, the database management system is MariaDB (MySQL), which uses Aria and XtraDB and in turn incorporates two other engines: PBXT and FederatedX. It also incorporates new system-level tables, which help in database optimization tasks thanks to the storage of service statistics.

The rest of the backend was built with Java and Spring framework. Furthermore, an application programming interface (API) was enabled to interact with the modules stored on the server, mainly built with Python 3.7. This API launches the algorithm library that is necessary for certain functionalities as the diet generator and returns the information in a JSON format to be stored and presented.

### 2.3. Sources of Data

This section contains the main sources of data that the APP needs in order to work. Moreover, several datasets were added to improve some of the functionalities and the user–APP interaction.

There are 5 main sources of information, with an additional source that incorporates useful data for the user. Other data sources could interact with the ones in this section, but they are module-dependent in the sense that they are mostly generated/used by the modules. The 5 main sources are:

1. User data: We store the main aspects of the user in a MySQL database, including information about biometrics, restrictions and behavior. This information allows us to calculate the nutrient levels we are aiming to recommend. At the same time, it also lets us filter several items in the dietary database that are not suited for the user, either due to age (as coffee or tea recipes in children) or to food allergies (not recommending milk-based products to users allergic to milk proteins). Other than hard restrictions, we also allow the users to check whether they dislike or like specific products and recipes in the dataset. The presence of preferences allows us to choose one recipe over the other when suggesting the menu. In this section, the user’s clinical history is also considered. Currently only the weight section (normal weight, overweight and obesity) and the allergy and intolerances are operational. This section can also take into account pathologies such as diabetes and hypertension. However, these options are currently inoperative as these options are not necessary for the target population of the nutritional interventions performed along the Stance4Health research project.
2. Nutritional references: These data are summarized in tables that are used as rules to generate a healthy menu. They contain recommendations for a Mediterranean and sustainable diet [26]. The EU Dietary Reference Values (DRVs) are the reference values on which the nutritional recommendations of the APP are based [27]. This module, therefore, establishes basic rules such as portions of food groups needed per week, or micronutrient intake for a healthy diet.
3. Ingredients data: The S4H APP contains multiple nutrient information from many stakeholders. Moreover, some of these data could be updated as new analysis and reviews are made. For that reason, nutritional information for every single ingredient is stored as another set of MySQL tables. Specifically, for this APP, we used the S4H food composition database (FCDB) developed within the framework of the project [28]. In summary, the S4H FCDB consists of more than 2600 foods with nutritional information on approximately 880 elements, including bioactive compounds. However, multiple elements can differ from one country to another. Moreover, several products are not consumed raw, but they undergo some kind of thermal processing. For that reason, our dataset contains a Branded Food Products Database consisting of food from supermarkets and hypermarkets of different countries (this is likely to be a significant percentage of the food already purchased and consumed by consumers). We specifically have detailed data from three different countries: Spain (with 89,385 foods products) provided by AECOC (Spanish Association of Manufacturers and Distributors), Germany (with 211,014 foods products) provided by ATRIFY and Greece (with 3312 foods products) provided by researchers [29]. We also included 670 different items from fast food restaurants obtained from the publications of the restaurant chains. These fast food items could be a recipe themselves, but as it is rare to solely eat one of them, we stored them as ingredients, to give the user more flexibility when entering the different menus which they could have eaten.
4. Recipe data: S4H APP, unlike recent approaches in food recommendations, follows recipe-centered meal planning. This means that our system recommends to the user a specific recipe for a specific time of the day. Unlike a single combination of ingredients, recipes give ingredients a context/relationship and a procedure. This allows the user to know “what to eat” and “how to cook it”. This selection has its drawbacks, too, which are analyzed in the discussion section. The recipes were reviewed by inhabitants from each country: it started with the analysis of more than 150,000 recipes from all countries to obtain a set of some 20,000 appropriate recipes (in terms of nutritional value, cultural traditions and diversity in all the possible meal plans). This dataset of recipes was then evaluated in terms of its ingredients’ names, weight, retention factors and yield factors according to the cooking technique described for each recipe. Finally, we obtained the nutritional composition of each recipe [28]. Additionally, users can create their own recipes, with ingredients from the ingredients database. Those users’ recipes will only be available for the users that have created them.
5. Expert knowledge: Despite recent trends in computational nutrition (that aims to provide automatic recommendations), having a source of expert knowledge has been proven as an excellent way to manage/rank all the nutritional goals and levels. Within this source, the APP includes:

- Food constraints related to health issues.
- Food levels related to age and biometrics.
- Food patterns that ensure a diverse diet.
- How several factors affect the previous items such as meal distribution, physical exercise and portion size.

Both physical exercise management and size management procedures have several novelty characteristics, thus they are explained in detail below.

### 2.4. Physical Activity

Daily physical activity can deeply affect the recommended nutrients. In the S4H APP, we addressed these variations asking the users to include a detailed description of their daily physical activity.

In order to calculate the metabolic expenditure related with physical activity, we established 5 groups according to the intensity of the activity. Then it is up to the user to provide the time expended along the day (in hours) in each category.

Activity values were assigned to each group of activities according to the Food and Agriculture Organization of United Nations (FAO) recommendations on physical activity level [30]. These values are weighted in the number of hours stated by the user, so that the daily physical activity is estimated. All these estimations are based on the guidelines proposed by the FAO [30]. The guide contains extensive information for children, adults and other physiological states, such as pregnancy and lactation.

### 2.5. Portion Sizes

Different sizes of standard meals were established. Each user chooses a meal size according to their preferences. Depending on the population group, the sizes range from XXS to XL. To standardize portion sizes (that is, link the weight of the recipe with the size letter) data were collected from 200 volunteers from different countries to unravel their usual consumption and how they viewed their portion, using photographic albums and pre-established portion sizes [31,32,33]. After that, we created a survey with Google Forms following the research model described in [34]. Each participant indicated the usual consumption size of the different food groups and dishes (i.e., a meat-based recipe, a fish-based recipe, a sandwich-based recipe, etc.). This was then compared with the consumption data and an average range was assigned. The results were used to classify the portion sizes in 6 different sizes (Figure 2).

### 2.6. Additional Data Sources: Images, Barcodes and User Interaction

Finally, there are other data sources which are not completely necessary but offer the user a better experience. These are the image data sources, the barcode information and the user interaction database. The image data source acts as a triple information service:

- Allows the user to see how a recipe looks when finished.
- Allows the user to identify ingredients easily.
- Allows the user to quickly check if they are using the correct product.

These three tasks allowed us to offer a better user experience, but could also be used in future studies assessing the impact of different imaging characteristics in food consumption/selection. It is worth noting that as we have two different sources of nutritional data (from recipes and ingredients), we then have two sources of imaging data: recipes and ingredients. Moreover, we also let the user upload images from their own recipes.

The barcode database allowed us to link several commercial products to their selected barcode. This information allows us to directly differentiate several products that could be really similar in terms of composition and visuals. However, it specifically helps in order to add an additional way to search for products, using the camera to process the barcode, which is then searched in the database.

In addition to the images’ data sources and barcodes, we also included a database for storing users’ interactions with the APP. This dataset contains data on users’ interactions in the APP, users’ changes, users’ skipped dishes, etc. This information could be used in future studies looking for users’ patterns of usage and behavior, but also for highlighting APP sections or mechanisms that should be changed to offer a better user experience.

### 2.7. Generator

From the aforementioned sources of data, we can run the dietary generator and add several modules that may modify how the generator works or what can be recommended. The main task of the generator is to combine the previous data sources and produce a dietary recommendation that fits nutritional levels with the other characteristics (meal size, preferences, meal behavior, health issues). The engine selected for this section follows the structure described in study [35].

### 2.8. Gut Microbiota Module

The gut microbiota module is a side-module that allows to make recommendations aiming to optimize the user’s gut microbiota. Despite the usual sources of nutritional information that can affect a meal recommendation, microbiota is rarely used despite its huge importance on overall health. For these reasons, we present this APP as one of the novel state-of-the-art recommendation engines that take into account the user’s microbiota to make recommendations.

Specifically, this APP aims to establish guidelines based on the interaction of the gut microbiota and the diet. Studies carried out within the S4H project developed the extended reconstruction of dietary metabolism in human gut microbiota AGREDA [36], which was subsequently improved using an enzyme promiscuity approach [37]. This network establishes relevant metabolic interactions between diet and the gut microbiota. Using 16S rRNA gene sequencing data from fecal samples, it is possible to establish interactions between microbiota species, evaluating the impact of different metabolites on them. We can then translate this impact into a score, which will affect the recommendation process and lead it towards a recommendation pattern that favors a set of microbial metabolite levels closer to the ones considered healthy. The information obtained provides a list of the overall gut microbiota score for each food. The score corresponds to a real number, which can be positive or negative depending on the impact of each food on the gut microbiota species. The generator focuses on recommending those combinations that are most favorable for the microbiota. This score can be effectively incorporated into the recommendation engine, favoring the most beneficial ingredients and minimizing the least favorable ones.

### 2.9. Search Engine

Giving flexibility to the user is key for them not to abandon the program. Moreover, it allows the user to describe what their daily food habits really are, which could be used to understand how they are deviating from the APP recommendation or other healthy markers. Finally, it also allows the user to make better buying decisions allowing them to see the nutritional information of commercial products in a simpler and visual way. For these reasons, two aspects are necessary: a way to search for recipes, and a way to find ingredients to create new recipes. Therefore, we incorporated several ways to accommodate these needs: text-, voice- and camera-based interactions:

- Camera-based interactions were primarily developed to allow users to have a quick interaction with commercial products as they may be the main source of deviation from the diet. This can be achieved through a comprehensive database linked to the commercial barcodes in the system. A user can easily scan a barcode with their smartphone and automatically find the product data in the APP database.
- Text-based interactions are based on similarity metrics of the text-chains introduced.
- Voice-based interaction runs on Google Voice recognition API.

It is also worth noting that recipes will be displayed with their information by 100 g if they are searched without context. However, on the menu screen, these recipes will be expressed as g/portion size, which offers a more realistic view of what they are eating.

### 2.10. Wearable

Wearables check and store biometric data. Specifically, we were interested in wearables able to monitor body fat mass and body fat-free mass, and that could be incorporated into the system with a raw software development kit (SDK). 

Through the SDK, the wearable module can connect with the APP and allows biometric data to be imported for a better adjustment of the user’s profile. This wearable also gives readings of blood pressure, sleep quality, steps or energy expenditure. It also measures body fat percentage by means of bioimpedance [38]. Since a wearable’s accuracy can be affected by various factors, their data are compared with data obtained through questions from the APP’s configuration screens. If there are no major differences, the data from the wearables will be considered valid and will be used for energy expenditure calculation. This module has room for improvement, and a recent aggregator of fitness wearables such as Google Fit could be integrated into it.

### 2.11. Shopping List and GPS Module

One of the key aspects of every meal plan is the shopping process. This process can be influenced by several factors such as personal preferences or market selection. Moreover, recommending a diet based on a recipe’s selection can produce some confusion in the user, as some of the recipe names and photos may not reveal the ingredients needed for its consumption. Therefore, we built another module that helps the user in buying the necessary ingredients.

First, for every generated menu, this module compiles all the recipes and produces a unified shopping list that aggregates the necessary ingredients in weekly planning, and gives the total amount that the user will need of each ingredient. The shopping list can also be updated by crossing out those that are already in stock or that have been bought. This section integrates the Google Maps service, which allows global positioning system (GPS) technology to provide the user with information about the food environment. This can facilitate food shopping by showing the nearest supermarkets and grocery shops.

### 2.12. APP Testing and Validation

There are several scales to assess the quality of health-related applications objectively and reliably such as the Mobile APP Rating Scale (MARS) [39] or the Nutrition APP Quality Evaluation AQEL scale [40]. MARS is one of the most widely used scales and has a User Version (uMARS) [41]. uMARS has 4 criteria: engagement, functionality, aesthetics and information. Each can be rated between 1 and 5, and then the average scores of the domains are obtained. Finally, an overall average score is obtained, which is indicative of the quality of the application.

To nutritionally validate the APP, different dietary records were used to study food consumption and the nutritional quality of the diet [42]. We included a food consumption frequency questionnaire (FFQ) and a 24 h recall (which was used for two non-consecutive weekdays and one weekend day). All questionnaires (uMARS, FFQ and 24 h recall) were used in online format by Google Forms. These forms were adapted to collect information for research purposes [34].

The APP was pre-tested for reliability to ensure that users do not experience any problems during the nutritional intervention. Throughout the test, the load, recalculation and processing speed tests were performed.

In addition, 20 people from different countries (Spain, Italy, Germany and Greece) aged between 12 and 60 years old tested the APP and filled in the uMARS scale [41]. These volunteers were recruited from the different research centers involved in the European project Stance4Health. Subsequently, 20 volunteers (19–25 years old, belonging to the Nutrition’s Bachelor’s Degree at the University of Granada, Spain) collected their food consumption using a 24 h dietary record during several days and an FFQ to assess whether the menus generated matched the dietary guidelines and what the users consumed. All people participated in a voluntary way and consented to the use of their data for research purposes.

### 2.13. Security and Ethical Aspects of APP

The legal and ethical issues of data protection and privacy in the context of personalized nutrition are extensive [2]. S4H APP guarantees that all the information collected from users is exclusively for the purpose of providing a better user experience in the use of the APP, as well as being used for research purposes. The data and results derived from the study may be published and presented anonymously in journals, conferences, etc., as indicated in the current regulation (EU) 2016/679 and the GDPR Directive 95/46/EC [43]. Furthermore, the ethical guidelines of the Declaration of Helsinki were followed.

The study was approved by different ethical committees and the nutritional intervention in which the APP is being used is registered under the number SRCTN63745549 [25]. Users must also give their consent and may request the elimination of all their data at any time. The APP guarantees, at all times, the anonymity of the user’s data.

In terms of security, the APP requires authentication by the user. An OAuth 2 authorization process was established. During the intervention, in order to access the APP, the user needs to enter a 10 character alphanumeric code which is provided by the researchers. This code is different for each country and user, and is the one that will load the specific data according to the needs of the study (e.g., supplementation information). All notifications, requirements and petitions that the APP requests to the users are printable. All information is encrypted and transmitted through SSL channels.

### 2.14. Statistical Analysis

The Python 3.7 module was used as an analysis tool, using the libraries of pandas [44,45] and SciPy (for calculations) [46], seaborn [47] and matplotlib (for final plots) [48].

We ran our analyses with three main goals. Our first step was to check whether the information from the users and the FFQ was aligned to the users’ biometric measures. After that, we produced a set of descriptive statistical metrics to check how different the levels were from the FFQ and the generated menus (around the kcal and macronutrients levels). All the data were plotted in different degrees of precision (box plot for distribution comparison and scatter plot for dot-to-dot comparison).

From the FFQ data, an average of consumption across days for every user and a set of micronutrients was developed. This consumption levels were corrected with the biometric data stated by the user (height, weight, physical activity). Consequently, this also produced a biometric profile for the APP, which meant that we obtained the different values that the APP should reach for every user. 

We ran a set of generations where we obtained the nutritional data (from a set of micronutrients) and analyzed their averages across two weeks. Both values were checked (in terms of minimal DRVs) and evaluated, selecting the average consumption for every user. FFQ data and generated data were then compared by the average consumption across the days. The differences on calories and macronutrients were tested for normality using a Shapiro–Wilk test. For those which passed the test (possibly normally distributed), we considered mean/average and standard deviation as being representative enough. We started calculating the differences for every user and micronutrient between APP and FFQ levels using the formula in (1).

(1) $${\mathrm{Diference}}_{\mathrm{App}-\mathrm{FFQ}}\left(\mathrm{Nutrient},\mathrm{User}\right)={\mathrm{Nutrientlevel}}_{\mathrm{App}}\left(\mathrm{User}\right)-{\mathrm{Nutrientlevel}}_{\mathrm{FFQ}}\left(\mathrm{User}\right)$$

As most users in the last study had the same amount of minimal DRVs, we then aggregated the data visually in a box plot. Comparison plots were made using the pandas boxplot functionality [44,45]: the box extends from the quartile values of the data Q1 (the value under which 25% of data points were found when they were arranged in increasing order) to Q3 (the value under which 75% of data points were found when arranged in increasing order) quartile values of the data, with a line at the median (Q2). The whiskers extend from the edges of the box to show the range of the data. They extend no more than from the edges of the box, ending at the farthest data point within that interval. Outliers are plotted as separate dots. These plots let us compare the full distribution of data side-to-side in a descriptive/qualitative manner. In addition, these distributions were again tested for normality using the Shapiro–Wilk test. As there were differences in their distribution, we decided to use a Kolmogorov–Smirnov (non-parametric) test to check whether they were different enough to highlight those levels that suffer from a huge variation between FFQ and APP.

## 3. Results

The APP is not only limited to propose healthy menus, but it also aims to increase the user’s knowledge and awareness of their nutritional choices. The APP works as a personal dietician. If the user misses a meal or changes recipes, the APP can re-adapt the menu. This process recalculates how much the menu has changed in terms of macro- and micronutrients and offers an alternative menu that incorporates those changes to some extent (+/− 30% of difference divided in 5 or more consecutive days).

Adding recipes allows a higher level of personalization, being able to give nutritional information, which makes the user aware of the foods he/she eats and how they affect their nutritional status. The APP includes daily tips on nutrition, food or physical activity to teach the user new elements about food and nutrition. There are also alerts that show the user if his/her behavior has had an impact on a set of relevant nutrients and how to fix them in the future.

### 3.1. Integration between Modules

**Figure 3:**
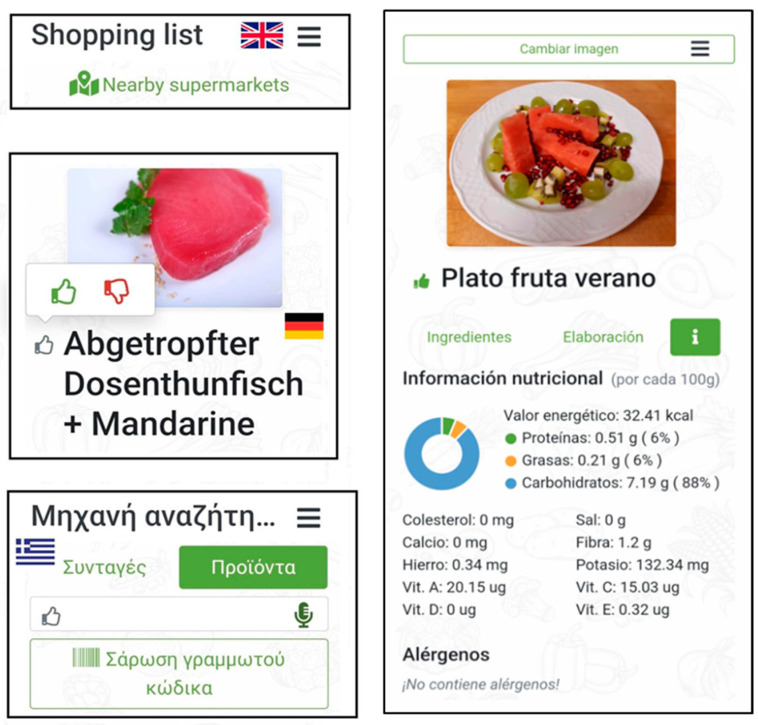
APP screenshots in four languages and different use levels. The four different languages used in the app are shown: left images, top (English), middle (German), bottom (Greek), right image (Spanish).

All modules of the APP were correctly connected to each other or executed independently without issues. The APP was tested in all languages and showed no problems (Figure 3). We added a few remarks on the interconnection of some of the modules.

Four modules were working interconnected in these trials: diet generation, preferences, microbiota and wearable. The diet generation process worked correctly, considering the rules addressed by experts, the nutritional information from the recipes and the user data. Although this module could produce a menu alone, the wearable, preferences and microbiota modules were integrated in the process and were able to modify the generating algorithm. These changes were used to modify and upgrade the menu score of the daily diet, achieving their secondary goal, which would be a better microbiome score, a higher presence of liked recipes and re-evaluation of energy consumption based on a wearable’s records. As the menu changes through different modifications, the recalculation in the generation module allowed reaching the healthy diet targets. Independently, other modules were activated. The GPS and camera modules worked properly (Figure 3). It was possible to connect and create recipes that could be integrated into the menu (Figure 3), which again represented a proper functioning both on frontend and backend, being able to communicate one to another, to store information in the dataset and in gathering/showing information to the user.

Menus were calculated for all possible situations that could arise during the nutritional intervention. Users of all ages with food allergies and intolerances were also tested. The results were optimal, but when using commercial foods, there were some challenges due to the absence of micronutrient information; in this case, the absence of micronutrient levels was computed as 0 in terms of quantity.

### 3.2. Testing the APP with Real Subjects

The APP was used by different team members, collaborators and test subjects in user mode. During the continuous stress and performance testing of the server, no problems were found, except for one that was due to server downtime. The APP works in an asynchronous way to reduce the loading times, which means that usually any change is stored in the server and performed independently. In these cases, the APP notifies the user that a change is going to be made, then the modification is performed in the server, uploading the results as soon as they are ready. Taking all this information into account, the spent time for a whole weekly menu calculation on the server-side was 24 s +/− 1.

**Figure 4:**
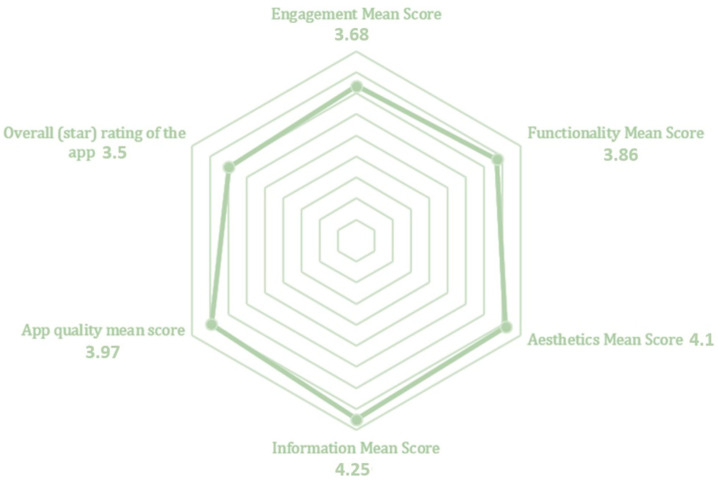
Average score for each section and user perception of the uMARS questionnaire.

The results of the uMARS questionnaires are depicted in Figure 4. The most valued by users was the information (with a score of 4.25/5), giving importance to the quality and credibility of the information included in the APP. Aesthetic appeal was the second most important and engagement was in last place (with a score of 3.68/5). Overall, the average quality score of the APP from users was 3.97/5. In addition, 88% of users would recommend the APP. Furthermore, 80% also reported that the use of the APP increased their nutritional literacy.

Menus were generated for 20 subjects and compared with dietary records from several days. To generate the menu, APP users’ keys were created for every participant. Those 20 subjects were then analyzed and we attained a set of goals and their levels from the generator. We noticed at the start of the test that all participants had different levels of micronutrient patterns, but overall, these patterns were aligned with the minimum DRVs. On the contrary, most of the macronutrient consumption was hard to align with the patterns from the APP. We divided the reasons for that into two groups: those participants that followed a hypercaloric diet with physical/fitness goals in mind, and those volunteers that have an unlikely eating pattern. 

Table 1 displays the differences between the data obtained by the APP and those obtained by the FFQ. All values except carbohydrates passed the normality test. For completion, we stated the values of the carbohydrates’ differences skewness: −1.53 and the kurtosis: 2.30. When removing 2 participants that eat an abnormal amount of carbohydrates, carbohydrates’ differences distribution passed the Shapiro–Wilk test with a skewness of −0.3 and a kurtosis of −0.09.

**Figure 5:**
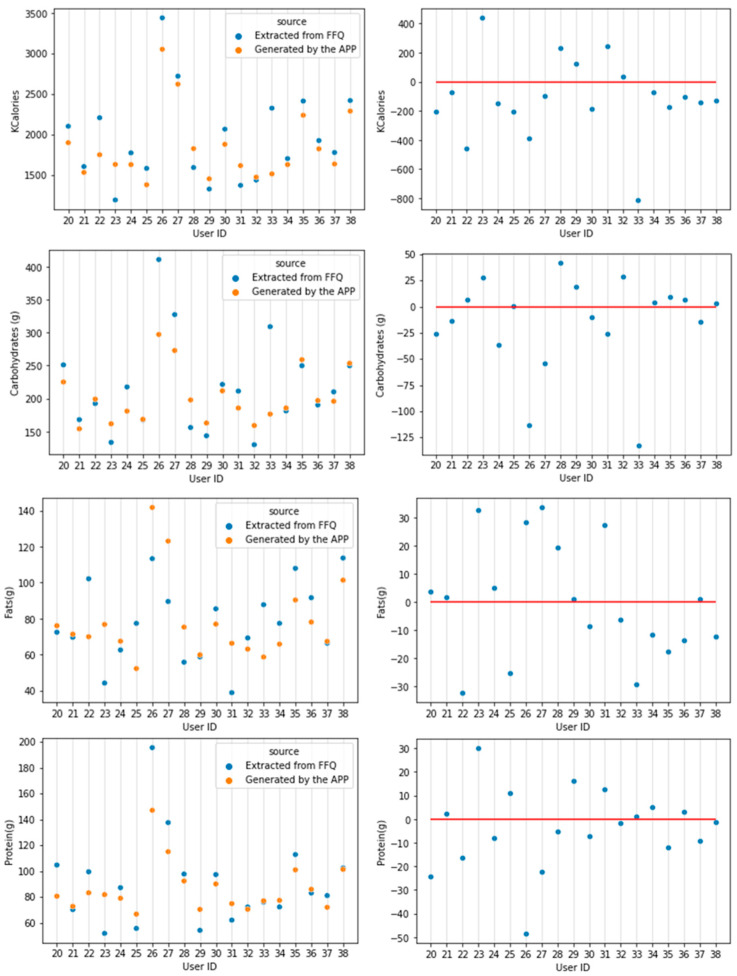
Energy and macronutrient comparisons from APP vs. FFQ (**left**) and differences APP-FFQ (**right**). Positive values mean APP levels were higher; negative values imply APP levels were lower.

On average, there was a global trend in the APP lowering the macronutrient intake of participants, which was mostly due to lower requirements in Kcal consumption (Figure 5). This trend varies in a large extent between users, specifically on Kcal consumption. However, most of the changes keep close to what FFQ suggested, except those subjects who had a high deviation from the expected level of macronutrients. A more detailed descriptive result can be seen in Figure 5 where every value is shown.

As expected, the rules of the generator offered a healthier macronutrient pattern consumption along with its goal of maintaining healthy habits over time. This also resulted in less variation of these quantities between days. 

**Figure 6:**
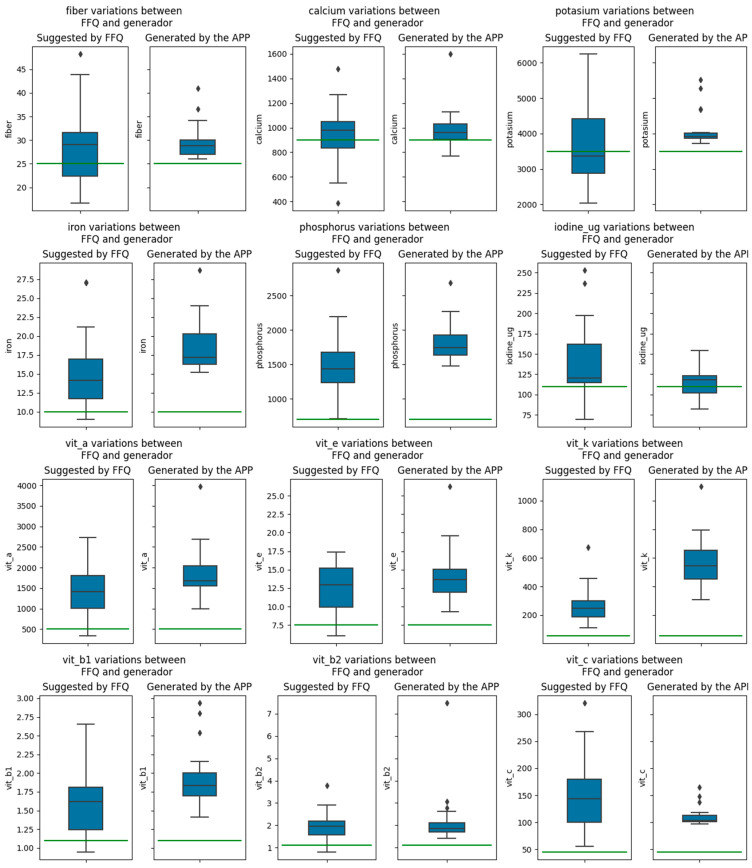
Energy and macronutrient comparisons from APP vs. FFQ. Green line specifies the inferior value of the DRVs.

On the micronutrient level, the generator also followed the DRVs in micronutrient levels. Results from the Kolmogorov–Smirnov test showed relevant differences in potassium, iron, phosphorus and vitamin K. The others were found to be statistically similar. However, even in those that were similar, the generator improved the micronutrient levels at the user level and remained within the DRVs in the rest. It also provided less differences between users as most of the levels of goals were the same, as can be seen in a smaller box in Figure 6 (all participants were in the range of 19–25 years old). Some levels of micronutrients (i.e., vitamin C) were higher in the FFQ data. Despite being lower, generator levels stayed within the DRVs. Iodine and calcium were the only micronutrients that stayed below the minimum levels recommended for some users. The whole data from the micronutrient comparison are depicted in Figure 6.

## 4. Discussion

The aim of this work was to show the development and validation of the S4H APP for a nutritional intervention. The APP was developed based on broad scientific support and tested by users. The results confirm that S4H APP was able to consider the habits and lifestyle parameters that users need to describe their eating habits, creating a high level of personalization. The APP information and interactions provided nutritional education to the users. This could also have a positive impact in the adherence and use time of the APP by the users.

The APP was able to generate and recalculate a healthy menu according to DVRs and reference dietary patterns. The correlation of nutrients with different dietary records demonstrates that the APP can be a reliable tool. The only difference detected was found for some users in iodine and calcium levels. Iodine could easily be managed by the system by adding some iodized salt to a few recipes. In the case of calcium, low levels were associated with a specific user and this was solved by swapping recipes for breakfast to include more dairy products. Another interesting outcome of the test was that the micronutrient levels of every user tended to stay above the DRVs; we did not observe as many variations as those obtained from FFQ, implying that the APP has a regulatory effect that will diminish differences in the daily basis of the users, while recommending to all of them the specific amount of macronutrients. The APP works with the constant evaluation of 12 micronutrients, but we also seek to expand this list in order to reach a nutritional evaluation process that includes a larger variety of micronutrients.

The modular development of the app allows to incorporate or to disable specific modules and to modify the generating algorithm, as in the case of the use of microbiota and/or wearable modules. These modules were being tested in a large human nutritional intervention (with data from fecal samples and wearables). For all these reasons, we believe that S4H APP will be a valid tool to support standalone personalized nutrition and to be useful in nutritional interventions, to incorporate several factors while offering a flexible healthy menu. 

### 4.1. Comparison of S4H APP with the Current Situation

The future of personalized nutrition involves challenging the great heterogeneity of individuals’ responses to diet and customizing nutrition according to each person’s needs [6]. Usually, nutritional applications focus on independent functions, such as building a food plan based on the user’s objective, generating a shopping list for the target diet, or ranking foods by scores [17]. The S4H APP has a modular design which provides a higher degree of personalization, thus efficiently unifying all the services in the same APP. While health- and nutrition-related APPs are increasingly used, few applications are evaluated and validated at the research level [1,11,17,49]. The popularity of nutrition-related APPs among healthcare professionals is also not particularly high [8]. Even so, studies have shown that dietary recording APPs are as valid as traditional methods of dietary assessment [20,50]. Although controversial, there is evidence that mobile nutrition APPs have great potential in nutrition research as well as the development of nutrition interventions [17]. In several cases, nutrition applications have improved results in terms of users’ knowledge and behavior [16,51,52]. In addition, APPs reduce the time needed for researchers to prepare data compared to traditional methods [17]. Some mHealth APPs incorporate elements of gamification or goal alerts, but expert recommendations are not provided [9].

In our case, the S4H APP provides expert-generated dietary and health guidelines aimed at increasing subjects’ nutritional knowledge and adherence. In parallel, several videos were created to show how to use the APP. The videos can be accessed from the APP itself.

Adherence to the nutritional plan is key in nutritional interventions. Involving users can be a constant barrier to prove the effectiveness of interventions, particularly in child nutrition [3,23]. For this reason, and in order to validate the results, the S4H APP was tested before the nutritional intervention to make sure that it was easy to use and to avoid drop-outs for that reason.

Aspects of the frontend were redesigned, adapting them to the latest trends, without losing the ease of use. Some volunteers reported that the use of the APP was time consuming, which has been already found in other studies [13]. Features were implemented to reduce usage time and give users more versatility. For example, we included quick functions to replace a proposed recipe with another one, to indicate that an intake has been skipped, or a button to go to the APP menu home instead of back. During APP testing, it was observed that breakfasts generated the highest number of recalculations. Therefore, an option was added where breakfast could be prefixed and excluded from recommendations and recalculations, thus speeding up the process. In addition, S4H APP has increased the number of alerts as many users forgot to change weight or modify menus. This is consistent with other studies where some participants stopped using an APP because they forgot to use it [12]. Studies have shown that mHealth APPs have a user adherence limit of 30 days [17]. Related to this, the S4H APP aims to provide all functions in a customizable way adapted to individual needs and objectives. According to König et al. [12], a high level of customization of the APP preserves the autonomy of the users and could extend its use. Increased utilization or a particular focus of the application could also lead to better results [53,54]. In this sense, the generator system was improved by incorporating aspects such as the seasonality of fruits and vegetables, the choice of bread and beverages during meals, or the creation of new rules to take care of gastronomic aspects (such as the repetition of recipes).

Having credible, evidence-based nutritional information also improves the user experience. The S4H APP is based on the professional nutrition software i-Diet [55], which is one of the most used nutritional software in Spain for more than nine years by nutritionists; this means that the development of the APP was based on previous nutrition experience. Some studies compared the validity of the use of nutrition APPs with diverse dietary analyses of food recordings [21,42]. Using different statistical tests, the results obtained from these applications were similar with the use of widely accepted and professionally used nutritional analysis software [21,42]. Thus, the S4H APP has a good correlation with dietary recording methods except for some micronutrients, whose reference values were not reached [27]. 

To strengthen the menu generation and to reach all recommendations, more recipes were introduced. In addition, branded products suffered a matching process similar to that described by other researchers [56,57]. This allowed to fill in data gaps for some nutrients in branded products, especially in fresh foods. This improved recalculation when using commercial products for recipe elaboration. A critical issue could be the use of inexact FCDB [1,12,14,20,58]. The approximate number of foods in the FCDB may be precarious or may have many missing nutrient values, and could underestimate macronutrient and micronutrient values [49]. In this sense, the FCDB used by the S4H APP was built in the framework of the European project Stance4Health [28], being currently one of the most complete FCDB worldwide in terms of nutrients and bioactive compounds.

Finding the right foods or recipes can also be a preoccupation for users [12]. The S4H APP enhanced the multilingual service by creating a standardized and comprehensive food corpus, making it possible to search for food and recipes in all four languages more efficiently. It is important to note that most academic food-related applications are usually only available in one or two languages at the most [49]. The S4H APP also offers a broad variety of recipes, which makes it attractive for discovering new dishes or food combinations. Recipes are often not taken into account for nutrient estimations [13] and are only used for meal planning or creation of shopping lists to reduce the associated time burden [50]. In the S4H APP, the recipes are essential in the generation of menus. In addition, the APP shows the proposed menu and the shopping list for the whole week, so that users can organize themselves. Currently, few APPs are able to propose or filter recipes according to food allergies and intolerances [59]. In this sense, the S4H APP modified their pre-existing recipes to generate recipes with alternative allergen-free foods that still meet nutritional requirements. The serving size provided by the APPs or the possibility to choose a food from the list of uploaded foods, especially recipes, are other aspects that can lead to more estimation errors [12,14,58]. There are currently several technologies and sensors used to estimate the quantity and serving size (such as cameras) to generate 3D images for food volume estimation, or photographs and videos captured by users and translated into nutrient content estimations [19,60]. The S4H APP was based on portion size measurements obtained from user feedback, facilitating the choice of these portion sizes through images. These tools have been used in other studies for many years [57].

Higher personalization makes the APP more attractive to users, which in our case is highlighted with the gut microbiota module and wearables. The use of later global positioning system (GPS) or cameras allows to take into account much more parameters in research, and to make the APP more attractive to users [15]. The gut microbiota composition and functionality is modified by diet [6,49], so that it is one of the new targets of personalized nutrition [61]. In this sense, the PREDICT I study evaluated the contribution of dietary context, metabolic and metagenomics contribution to gut microbiota responses to predict individual responses to foods through machine learning algorithms [62]. Taking all this information into account, the Stance4Health project has focused on the relationship between diet and how it affects the gut microbiota [36,37,63]. These data have been extrapolated to the S4H APP, providing an increased personalization in the generation of menus. Moreover, the diet proposed by the S4H APP is adaptable and capable of modulation according to the results of gut microbiota interactions. Currently, only the ZOE APP takes into account the gut microbiota composition [64]. Users appreciated a high level of personalization of that APP, although it is currently only available to a small group of consumers at a high prize [2]. According to recent studies, only a few users were willing to pay for personalized nutrition services [3,7,13]. Therefore, the S4H APP will be free of charge for those volunteers participating in the nutritional intervention. 

One of the limitations of the APP testing is that currently only a small part of the clinical history of the user is taken into account, in particular, weight, allergies and intolerances. This is due to the population targeting of nutritional interventions of the Stance4Health project. In the future, the APP could be adapted to other clinical information (such as pathologies like diabetes or hypertension) or to specific dietary patterns (like vegan diets). Finally, another of the strengths of the S4H APP is the modular structure. Further modules could be implemented in the future, such as sustainability or the economic value of the diet. This could bring more personalization.

### 4.2. S4H APP Security and Quality

Ensuring that data are stored securely and that privacy is respected are key factors for users of mHealth [12]. One example was the concern over a data breach at one of the world’s leading diet monitoring applications [49]. The European Commission, as well as governmental agencies, have created recommendations on the design, quality and safety of these applications [65]. The S4H APP includes all the necessary security parameters to guarantee secure and anonymous use. Indeed, some APPs implement social media options [9]. In the Stance4Health study, these options were not implemented in order to guarantee the anonymity of the subjects participating in the nutritional intervention. The only incident during testing was the server downtime. Thus, the S4H APP established complementary actions to avoid future problems with the server.

According to Bzikowska-Jura et al. [1], a nutrition APP must have certain minimum parameters to be a suitable application. Although there are many tools for assessing the quality of APPs, the MARS scale is the most commonly used [3,22,39,59]. According to the different studies that evaluate the quality of nutrition APPs, the average quality of Mars scales ranges between 3.1–3.8 out of 5 points [3,22,39,59]. Top-rated APPs in the respective online shops received low MARS scores, reflecting poor quality. Thus, the number of reviews per APP may not always reflect quality [5]. In our study, we focused on the users’ opinions, so we used an adaptation of the MARS scale for users, uMARS [41]. On the uMARS scale, almost 80% of the nutrition-related APPs scored above 3 out of 5, with a median score of 3.5 [4]. Our test results were higher, especially in the area of information. This indicates that users value all the scientific work and effort conducted during the APP development.

## 5. Conclusions

The global goal of the Stance4Health project is to develop a complete Smart Personalized Nutrition (SPN) service based on the use of mobile technologies, as well as to optimize the gut microbiota activity and consumer engagement in the long term. 

We have shown that technological developments in recommender systems and the processing of medical records and user data will greatly improve the overall personalized nutrition landscape and application features. Being able to add more data sources will make it possible to identify and correctly act on more cases, making the application more inclusive. At the same time, this opens the challenge of effectively combining these sources into a recommendation. S4H APP is an example of this effective integration. We believe it is interesting to further explore which of these combinations are feasible, working on a multifactorial recommendation. 

Microservices architecture is another fundamental approach for these APPs. We have shown that S4H APP effectively uses this kind of structure. Long-term dietary recommendations remains a very interesting challenge that needs to combine many data sources. Although it may seem disconnected, the microservices architecture is fundamental in this part, as it could allow us to add ways to gamify the application. This would encourage the continued use of the APP. 

All these advances will provide (in the future of personalized nutrition) an interesting case study for the ethical aspects of the technologies. A correct processing of tastes, preferences and medical needs opens a field to explore a correct balance between all of them. In turn, extending personalization also means constantly dealing with patients’ clinical data but also with their dietary patterns. We believe it is necessary to expand research on this topic, and the S4H APP is a novel platform that can allow these kinds of studies.

In conclusion, the S4H APP is a step up in the combination of different data sources for personalized recommendation. It combines healthy parameters, user needs and novel dietary parameters as microbiomes or wearable data. It will be used as the main tool for the nutritional intervention carried out in the Stance4Health project, where the rest of the modules are under testing and we could further explore the future directions discussed. 

## Figures and Tables

**Table 1:** Differences between generated APP levels and those obtained from FFQ.

	Kcal	Carbohydrates (g)	Fats (g)	Proteins (g)
Average	−111.7	−14.6	−0.1	−4.0
Standard deviation	273.2	45.1	20.5	17.1

## Data Availability

Not applicable.
